# Sleep and circadian difficulties in schizophrenia: presentations, understanding, and treatment

**DOI:** 10.1017/S0033291725000297

**Published:** 2025-02-17

**Authors:** Daniel Freeman, Felicity Waite

**Affiliations:** 1 Department of Experimental Psychology, University of Oxford, Oxford, UK; 2 Oxford Health NHS Foundation Trust, Oxford, UK

**Keywords:** Sleep, insomnia, nightmares, circadian rhythms, psychosis, schizophrenia, delusions, hallucinations, treatment

## Abstract

It is common in mental health care to ask about people’s days but comparatively rare to ask about their nights. Most patients diagnosed with schizophrenia struggle at nighttime. The next-day effects can include a worsening of psychotic experiences, affective disturbances, and inactivity, which in turn affect the next night’s sleep. Objective and subjective cognitive abilities may be affected too. Patients commonly experience a mix of sleep difficulties in a night and across a week. These difficulties include trouble falling asleep, staying asleep, or sleeping at all; nightmares and other awakenings; poor-quality sleep; oversleeping; tiredness; sleeping at the wrong times; and problems establishing a regular sleep pattern. The patient group is also more vulnerable to obstructive sleep apnea and restless legs syndrome. We describe in this article how the complex presentation of non-respiratory sleep difficulties arises from variation across five factors: timing, mental state, need for sleep, self-care, and environment. We set out 10 illustrative patterns of such difficulties experienced by patients with non-affective psychosis. These sleep problems are eminently treatable with intensive psychological therapy delivered over approximately eight sessions. We describe key techniques and their typical order of implementation by presentation. Sleep problems are an important issue for patients. Giving them the therapeutic attention patients often desire brings both real clinical benefits and improves views of services. Treatment is also very likely to lessen psychotic experiences and mood disturbances while improving daytime functioning and quality of life. Tackling sleep difficulties can be a route toward the successful treatment of psychosis.

“It took a while to reset the sleeping pattern from crazy hours, nocturnal to a normal time. To shift the clock back. There are techniques taught that I will never forget. Yeah, my sleep now is perfect. When my sleep pattern changed over the period of three weeks, and I started to get eight hours solid, I would wake up feeling a different person, like I haven’t felt in probably a few years. I felt really great and there was less worry, less stress, less paranoia. It was lifting. Yeah, it makes a massive difference on feeling paranoid, if you have good sleep and how your sleep pattern is.”

“I’ve had periods where I have heard voices at night when I was trying to get to sleep. So they would prevent me from sleeping and make my insomnia, which I had anyway, it made my insomnia quite a lot worse. It kind of jolts you sometimes, I think, when I’m trying to get to sleep. And I’ll hear a voice say something in my head, and it’s like a jolt.”

“So what was stopping me from sleeping was just the rumination, the thoughts of, you know, doom and dread, you know, why people are doing, are doing, the things that they’ve done…You know, you’re starting to feel unwell during the day because you haven’t had enough sleep. So for example, you know, you can start to hallucinate.”

## Introduction

Partway through the Better Sleep Trial (BEST) (Freeman et al., 2015) – the first randomized controlled trial of a psychological sleep intervention for patients diagnosed with non-affective psychosis – we decided to characterize the typical sleep presentations of the participants. From the baseline assessment of the first 10 patients, we gathered the 7-day actigraphy data (from a wearable device that detects movement) and the sleep diaries. The main entry criteria for participants in the trial were simple: a diagnosis of non-affective psychosis, a current delusion and/or hallucination, and a score on a self-report questionnaire indicating potential insomnia. Yet, when we inspected the data, we found that none of those first 10 patients had the same sleep difficulties; each experienced quite different problems. In this article, we will provide a framework to help explain why there might be such heterogeneity in sleep and circadian difficulties in patients with psychosis. Yet, we will also characterize typical presentations and highlight key treatment techniques for each to help those treating such sleep difficulties. Treating sleep problems has been a low priority in clinical services for psychosis, but it is a high priority for many service attendees. This disjunct is largely the result of a historical belief that sleep difficulties are a consequence of psychosis. Our evidence, however, suggests that sleep problems are in fact a likely contributory causal factor for many difficulties experienced by people with psychosis. Hence, treating sleep disturbances may be an important step for patients to reduce psychotic experiences and associated difficulties. By recognizing the importance sleep problems have for so many patients, treatment may also increase satisfaction with services.

### Sleep and psychosis

Rates of sleep disturbance in patients with non-affective psychosis are clearly high (e.g., see Bagautdinova et al., 2023; Freeman, Taylor, Molodynski, & Waite, 2019; Lunsford-Avery, LeBourgeois, Gupta, & Mittal, 2015; Ma, Song, Xu, Tian, & Chang, 2018; Sheaves, Onwumere, Keen, Stahl, & Kuipers, 2015; Waite, Sheaves, Isham, Reeve, & Freeman, 2020). In a diagnostic interview study with 60 patients with early psychosis, 80% had a diagnosable sleep disorder, half of which were rated as severe, with an average of three disorders per person (Reeve, Sheaves, & Freeman, 2019). The most common sleep disorders were insomnia and nightmares. Likely at least partly due to side effects of antipsychotic medication, higher rates of sleep apnoea, via weight gain, and restless legs syndrome, via dopaminergic deficiency, are seen in patients with psychosis (Kalucy, Grunstein, Lambert, & Glozier, 2013; Kang et al., 2007; Weber et al., 2022; Winkelman, 2001). There is a separate literature on sleep and bipolar disorder (see Harvey, 2008; Kaplan, 2020; Tonon et al., 2024).

It is also clear that higher levels of sleep difficulties are associated with higher levels of psychotic experiences across nonclinical, ultra-high risk, and diagnosed populations (e.g., see Blanchard et al., 2020a; Clarke et al., 2021; Freeman, Pugh, Vorontsova, & Southgate, 2009, 2010; Koyanagi & Stickley, 2015; Lunsford-Avery et al., 2015). Sleep problems predict the occurrence of psychotic experiences (e.g., see Formica et al., 2024; Freeman et al., 2012; Kammerer, Mehl, Ludwig, & Lincoln, 2021; Reeve, Nickless, Sheaves, & Freeman, 2018; Sheaves et al., 2016; Wang et al., 2023; Waters, Chiu, Atkinson, & Blom, 2018). A key route by which insomnia influences psychotic experiences is negative affect (e.g., see Bagrowska, Pionke-Ubych, Majchrowicz, & Gawęda, 2022; Ballesio et al., 2022; Freeman et al., 2009; Freeman et al., 2010; Kasanova, Hajdúk, Thewissen, & Myin-Germeys, 2020; Rehman, Gumley, & Biello, 2018; Scott, Rowse, & Webb, 2017). From our clinical experience, we believe that sleep disturbances may be a causal contributory factor in the occurrence and maintenance of psychotic experiences and therefore that treating these disturbances should lessen the psychotic experiences (Freeman et al., 2009; Myers, Startup, & Freeman, 2011). In a randomized controlled trial of 3,755 university students with insomnia, treating sleep difficulties significantly reduced (nonclinical) paranoia and hallucinations (Freeman et al., 2017). Psychotic experiences and insomnia share genetic and environmental influences (Reed, Jones, Hemani, Zammit, & Davis, 2019; Taylor, Gregory, Freeman, & Ronald, 2015) (i.e., they can both arise from the same causes) and likely have a reciprocal relationship in exacerbating each other. However, in our view, the predominant route is for sleep difficulties to contribute to the occurrence of psychotic experiences.

Poorer quality of sleep in patients with psychosis is also associated with lower quality of life (Ritsner et al., 2004; Ong et al., 2020), potentially poorer cognitive performance (Carruthers, Brunetti, & Rossell, 2021; Kimhy et al., 2022), and poorer daily functioning (Blanchard et al., 2020b; Stafford, Oduola, & Reeve, 2024). Sometimes, patients simply cannot get up in the morning to engage in education or work. The implication is that treating sleep difficulties could bring a wide range of other benefits for patients in its wake.

### Why are sleep presentations so varied?

“People that knew me would be like drive past my flat and the curtains are shut in the day because I’m sleeping. There’s a REM song, Day Sleeper. That was me. And it’s not good because we need sunlight. We need people. We need to see and talk to people. And if you’re awake at three or four in the morning, it’s not a good place to be.”

“The night terrors, so when I’m in paranoia, when I’m in psychosis, I have the night terrors every night. I try to avoid them, I try to do everything but I can’t and usually I’m highly medicated but it still doesn’t stop the night terrors and I’ll go to sleep and I wake up and I’m scared of opening my eyes…I just wake up, the night terrors I just wake up screaming, I have these certain scenarios that go on in my head.”

“When I was feeling most unsafe, my sleep was terrible. I would be, I would struggle to get to sleep on a night. I’d struggle to stay asleep on a night, I’d struggle to get up on a morning. So it just, it was just everything was wrong with it basically.”

The great variation in human personality can be ascribed to the multiple combinations possible across the five trait dimensions (extraversion, agreeableness, conscientiousness, neuroticism, and openness to experience) (Goldberg, 1990). An analogous case can be made to explain the variety of sleep presentations in psychosis. Drawing on the general factors that affect sleep (time spent awake [homeostatic load], time of day [circadian rhythm], and level of arousal) (Borbély, 1982; Riemann et al., 2022; Saper, Cano, & Scammell, 2005), our heuristic analysis of non-respiratory sleep problems focuses on variation across five sleep-relevant dimensions. Those dimensions are timing, mental state, need for sleep, self-care, and environment (see Table 1). *Timing* can vary by when a person goes to bed, when they fall asleep, the length of undisturbed sleep periods, when they wake up and when they get up, chronotype, age (phase delay in adolescence and phase advance in older adulthood), the regularity of sleep and wake times, and the degree of alignment to the day/night cycle. *Mental state* can vary, for example, by the level of anxiety, worry, and rumination at nighttime, the presence of psychotic experiences such as paranoia and hallucinations, the degree to which the person feels generally defeated and hopeless, the occurrence of nightmares and similar phenomena, experiences of pain, and the effect of medications. The *need for sleep* is most typically affected by the level of activity, employment demands, rest, and napping. *Self-care* pertains to the intake of food, drinks, and other substances, and their timings. Last, we also consider variations in the *environment*, including safety, light, electronic devices, comfort, the social environment, and living arrangements.

**Table 1:** Factors to consider when understanding a patient’s presentation of sleep difficulties

Dimension	Clinical questions
Timing	When did the person get into bed? When did they fall asleep? How long did they fall asleep for? Were there nighttime awakenings? When did they wake up? When did they get out of bed? How much sleep did they get? Is the sleep pattern consistent across a week? What would be their sleep timings if they had good quality sleep?
Mental state	Are they anxious, worried, or ruminating in the evenings? Do they have a wind-down routine? Are they worried about their safety at night? Are they experiencing hallucinations? Is the person having nightmares or bad dreams? Do they wake in the night feeling anxious? Are there any other unusual night-time experiences? Do they wake up gasping for breath? (potential sleep apnea check) Do they snore? (potential sleep apnoea check) Are they in pain? How is their motivation to get up for the day? What medications are they taking? Are they drowsy in the morning from medications?
Need for sleep	Is the person active enough during the day to be tired at night? Are they getting natural light in the morning? Are they napping during the day? Are there demands from work or other activities affecting their sleep?
Self-care	What is the person drinking and when during the day? Is the person having regular meals? When is the last meal of the day and what is it? Are they smoking, drinking alcohol, or using drugs?
Environment	Does the person have a bedroom and a bed that they are using? Is the environment dark during the night? Does the person feel safe in their home? Is there anyone else at home? Does the person play computer games or use other devices at night?

### Successful treatment of sleep and circadian difficulties in psychosis

“I found that if I’d spent the whole day not very active and hadn’t gone anywhere I wouldn’t be able to sleep very well and unwind. But me personally, I felt that if I had gone out on a bike ride or a bit of walking, bit of jogging or a bit of gardening, then that helps you unwind in the evening. So if you’re struggling with sleep and it’s easy for me to say, but try and be active in the day if you can and that will help in the evening. And I also remember learning to, you need to unwind in the evening because we’ve, we’ve had stimulation all day long. Um, and when you try and sleep, you’re still stimulated. So try and have a routine in the evening where you de wind and then go to bed.”

“We decided to do the 15-minute rule. If you’re not asleep within 15 minutes you get up, you come downstairs, you leave the bedroom alone. You don’t watch the telly, you don’t do anything, you don’t go on your computer, you don’t do anything that’s gonna rejig your head into thinking it’s get up time. Anyway I used to do dot to dot or word searches…That took a long time and I’m talking weeks not days I’m talking weeks maybe months. Now I can go to sleep in 15 minutes.”

“Well, in terms of my sleeping at the minute, I’ve recovered quite a lot. I’m able now to be able to, you know, go to sleep about one or two and have six to seven hours. At my worst, I wasn’t sleeping at all. I was literally having to take diazepam and sleeping pills, other sleeping pills, just to get about four or five hours sleep because I was so convinced that my life was coming to an end.”

Successful treatment of sleep problems means helping the person achieve consolidated, restorative sleep at the correct stage of the light–dark cycle. Short-term use of medication can have its place (De Crescenzo et al., 2022), and for obstructive sleep apnea continuous airway pressure devices may be needed (Giles et al., 2022). However, for most sleep disturbances in patients with psychosis, we recommend evidence-based psychological treatment such as cognitive behavior therapy. This is in line with guidance for the rest of the population (Baglioni et al., 2020; Edinger et al., 2021; Espie, 2021; Morin et al., 2024; Palagini et al., 2024; Riemann et al., 2017).

What should that treatment look like? Elsewhere we have described 10 principles for the design and development of effective psychological therapies for psychosis, such as our adapted sleep treatment. These principles should be perceptible in delivery. They are as follows: be respectful, be precise, target key mechanisms, frame positive counter-weights, allow for complexity, build in measurement, involve people with lived experience, build credibility, develop optimism, and plan for implementation (Freeman, 2024). The key mechanistic targets to improve sleep are those that contribute to its regulation: sleep pressure, hyperarousal, and circadian rhythms. Sleep pressure is built up during the day; nighttime hyperarousal is reduced; the bed is re-associated with sleep at the correct time of the day; and a plan is put in place for nighttime awakenings. The potential effects of the environment and self-care on these targets is kept in mind.

Intervention, although relatively brief, needs to be of high intensity for this patient group. Typically, we provide eight 1-h individual sessions over 12 weeks. However, we supplement this with between-session contact to help implement changes (e.g., telephone calls, text messages, and emails). We also provide therapy booklets. Home visits can be helpful to see the sleep environment. The therapist, or an assistant, may go along with the person to help initiate new activities. To ensure the intervention is working, the patient is asked to keep a sleep diary during the week and complete a sleep questionnaire at every session. Therapists have weekly supervision, in which the formulation is agreed upon, implementation of techniques discussed, and sleep diary and questionnaire scores reviewed. The sleep difficulty must be rapidly, but accurately, conceptualized so that time can be predominately spent on implementing the techniques. Implementation of a technique is conducted assiduously.

The core techniques comprise the following: setting an appropriate sleep window (with changes often implemented incrementally), increasing daytime activity levels, developing an evening wind-down routine and a morning rise-up routine, and stimulus control (e.g., only using the bed and bedroom for sleeping, getting out of bed if still awake 20–30 min after lights out, and stopping daytime naps where appropriate). As part of this work, more helpful circadian rhythms are re-established (e.g., using light as an entraining agent and establishing regular meals and activities). That is, circadian rhythms are considered in building up daytime patterns of activities. Other techniques are used depending on the specific presentation. These include nighttime strategies to deal with psychotic experiences, imagery rehearsal for nightmares, preparing for nighttime awakenings, sleep-facilitating behaviors (e.g., developing a suitable sleep environment and decreasing the use of caffeinated drinks), implementation of worry periods, helping patients not to use daytime sleep as a strategy to avoid voices (often a source of hypersomnia), and methods to desensitize to the bedroom when it has been associated with past trauma. We may work with others (e.g., parents, bed partner, and care team) to support the changes. We also work with prescribers and patients to consider the effects of medication. Most commonly, this involves finding the best time to take antipsychotic medications for sedation so that they help sleep rather than produce daytime grogginess. Cause and effect are unclear, but the severity of sleep difficulties differ by antipsychotic medication (Cederlöf et al., 2024), with stronger evidence that clozapine may lead to fewer insomnia symptoms (Miller, McEvoy, & McCall, 2023). Details of several of our first sleep treatment adaptations are provided elsewhere (Sheaves et al., 2018; Waite et al., 2016).

We have conducted case series evaluations of sleep interventions for patients with current persecutory delusions (Myers et al., 2011) and patients at ultra-high risk of psychosis (Bradley et al., 2018). These have led to small randomized controlled trials for people diagnosed with psychosis and current delusions and hallucinations (BEST) (Freeman et al., 2015), patients at acute episode admitted to psychiatric hospital (OWLS) (Sheaves, Freeman, et al., 2018), patients with current persecutory delusions and nightmares (NITES) (Sheaves et al., 2019), and patients at ultrahigh risk of psychosis (SleepWell) (Waite et al., 2023). The pattern of findings has been consistent. High-quality sleep intervention is popular with patients, with high uptake rates. Moreover, every evaluation has shown very large improvements in sleep. There is evidence, too, of improvement in other outcomes such as paranoia and psychological wellbeing. For example, in the SleepWell trial, 40 patients at ultra-high risk of psychosis were randomized to receive sleep therapy plus usual care or usual care alone (Waite et al., 2023). Compared to the usual care, the sleep intervention led to a very large effect size reduction in insomnia at the end of the treatment (Cohen’s *d* = 2.7), which was sustained at the 9-month follow-up (Cohen’s *d* = 1.9). There were also improvements in other outcomes such as anxiety, depression, and paranoia. Qualitative analyses support the positive outcome data from the clinical trials (Waite, Evans, et al., 2016; Waite et al., 2018; Waite et al., submitted).

Interesting convergences reflect clinical realities. Clinical researchers have also highlighted that most patients with sleep problems present with multiple difficulties (Sarfan, Hilmoe, Gumport, & Harvey, 2023). Harvey and colleagues (Harvey & Buysse, 2017; Harvey & Sarfan, 2024) therefore developed a protocol to treat many sleep problems across a range of mental health disorders over up to 12 sessions. The four core modules are as follows: establishing regular sleep–wake times with appropriate routines, improving daytime functioning to increase energy, challenging unhelpful beliefs about sleep, and planning how to maintain new behaviors. Seven optional modules – such as improving sleep efficiency and reducing time in bed – can be used flexibly. In a clinical trial, 121 adults with serious mental illness (defined as the presence for 12 months of at least one significant mental health disorder) and sleep difficulties were randomized to receive the transdiagnostic sleep intervention plus usual care or usual care alone. Schizophrenia spectrum disorders (49%), anxiety disorders (47%), substance-use disorders (34%), and major depressive disorder (29%) were the most common past or current diagnoses within the patient group (in which comorbidity was common). The most common sleep disorder was insomnia (82%). The average number of sleep sessions attended was eight. Compared to the control group from pre-treatment to post-treatment (9–14 weeks), the sleep intervention produced a large reduction in sleep disturbance (Cohen’s *d* = 1.0) that was maintained at 6 months (Cohen’s *d* = 0.8). A range of other benefits were also seen, both in terms of improved functioning and reduced psychiatric symptoms, including delusions and hallucinations. These results reinforce our belief that, when sufficient time is devoted to targeted sleep treatment, there are real grounds for clinical optimism.

### Ten typical presentations and their treatment

We have highlighted the great variety in the presentation of sleep difficulties in patients with psychosis. However, in treating these difficulties, it is helpful to be aware of common presentations and how to tackle them. When we train therapists now, we highlight 10 non-respiratory sleep presentations. We set these out below and highlight the key techniques often used. Nonetheless, an intervention will always follow an assessment to identify the most important factors for the individual. Furthermore, patients may also have several of these sleep types, which requires a decision – drawing on knowledge of the key sleep mechanisms – about the initial focus likely to lead to the most change. Typically, there needs to be persistence with implementation of the core sleep techniques, before any significant work on psychotic experiences, since the benefits may take a few weeks to become fully apparent.

*Type 1: Going to bed early and not sleeping.* Many patients head to bed early in the evening and this is understandable: they may not have much else to do, the bed can seem a place of comfort, and a habit has been formed. Going to bed early may also seem to offer the best chance of catching up on sleep. However, the best quality sleep generally occurs later in the evening, when tiredness has built up from a busy day. Going to bed early tends to lead to worry and rumination in bed, and potentially greater focus on hallucinatory experiences. The key treatment steps are adopting a later bedtime and finding things to do in the evening before starting a wind-down routine. It may be necessary to learn to associate bed with sleep by using the 20–30 min rule of getting out of bed if not sleeping (stimulus control) (Bootzin, 1972). This will all work better if the person implements a morning rise-up routine and is busier during the day. Strategies to shift attention away from hallucinations (such as voices) when in bed may be necessary.

*Type 2: Going to bed in the early hours.* Some patients do not think about going to sleep until the early hours of the morning. They might be on their phones or laptops, they might be playing games or working on projects, pottering around the house, or simply thinking. The day has not been put to rest. Sleep quality is often poor and the person may struggle to get up to do things during the day. Attendance at school, college, work, or appointments can be affected, as the person is out of step with the world. To develop a better sleep pattern, we often start with a consistent sleep schedule. This means getting to sleep and getting up at the same time every day for a week or two. To do that, we need to find the times that will be achievable for the person most days of the week. Once a regular sleep pattern has been put in place, we gradually move the getting up time earlier. Typically, we aim for a shift of half an hour over the course of a week. This change is supported by developing a really good rise-up routine. The person will eventually feel more tired earlier in the night and hence fall asleep earlier. Shifting a sleep pattern by 2–3 h will take several weeks.

*Type 3: Going to bed at around 10 pm*–*12 am and lying in bed for an hour or two not sleeping.* This is an intermediate presentation between the extremes of the first two types. Our therapeutic focus is the lying in bed, during which the person may be beset by worries, hallucinations, and frustration. The key step is to push back bedtime by an hour or two to reduce the time spent in bed without sleeping. The 20–30 min rule will probably be necessary to associate bed with sleeping. Developing a wind-down routine so that the person feels calm and relaxed before going to bed is also very helpful.

*Type 4: Chopping and changing sleep times: an inconsistent sleep pattern.* Sleep is more likely to come with a regular, predictable rhythm. Going to bed at the right time and the same time each night – and getting up at the right time and the same time each morning – is the foundation for training the body to sleep well. When this is absent, the first step is to identify what is currently happening by means of a sleep diary (Carney et al., 2012). It can also indicate when sleep is most likely to come in a good block. The next step is to set a sleep window – regular times to go to bed and get up. This window must be in sync with the day and night (in other words, the person needs to be awake and active in the day and get their sleep at night). It also needs to be of a length that matches how much a person typically sleeps. The way to help sleep happen in that window of opportunity is via the use of wind-down and rise-up routines and stimulus control techniques.

*Type 5: Little difference in activity between night and day.* Sometimes patients are simply getting little pockets of sleep through the day and the night. It might sometimes be on the sofa, in a comfortable chair, or on the bed. Between these times, they are often pottering around the house or watching television. They may sleep during the day to avoid voices or be active at night to avoid other people. There is little variation between what the person does at night and during the day – whether that be activity or sleep. When the person does sleep, the quality is poorer because the best sleep is consolidated at nighttime. Therefore, treatment needs to ensure that days and nights are different. The day must contain activities to help build up tiredness. That way, when the person does go to bed at nighttime, they are ready to sleep. If possible, naps are cut out or shortened (often by scheduling activity). Good anchors for the day – such as a rise-up time, meal times, key activities, and a wind-down routine – should be put in place.

*Type 6: Sleeping over 9 or 10 h a night and not feeling refreshed.* Excessive sleepiness or hypersomnia occurs in a significant minority of patients with psychosis (Reeve, Sheaves, & Freeman, 2021). Oversleeping is not healthy; moreover, it may do nothing to lessen feelings of tiredness, fatigue, or sleepiness. Dreams and nightmares are more likely. It can also prevent people from doing daytime activities. There are two interconnected parts to limit oversleeping and to feel less tired. A good amount of sleep is 7–9 h a night. Therefore, it is best to shorten the period of sleep, at least by an hour or two. This can be done in steps over a period of weeks. A realistic time to get up each day needs to be set and then implemented. Once a regular getting up time is achieved, it can be gradually pushed earlier, typically by half an hour at a time, established over days. Crucially, we need people to get up and be active during the day, irrespective of how tired and groggy they may feel at first. We need a strong rise-up routine and a good reason to get up. Therapy becomes focused on daytime activity.

*Type 7: Nightmares.* Bad dreams can make people fearful of going to sleep and, when sleep does eventually come, disturb that rest. First, we want to reduce the chances of having a nightmare in the first place. This means bringing calmness to the evening and taking steps to maximize the chances of good-quality sleep, such as a busy day, a good wind-down routine, and healthy food and drink intake. Second, we can also work directly on the dream content to take the sting out of it. This is called imagery rehearsal therapy (Krakow, Kellner, Pathak, & Lambert, 1995; Sheaves et al., 2019). It involves exploring, developing, and practicing new ways of finishing the storyline of the dream. By spending time gently going over the nightmare and finding new paths that the story can go down, it is possible to change the dream and/or the reaction to it. Two other factors should be kept in mind. Dreams are more likely if a person is oversleeping, so this may need addressing (see Type 6). Certain medications can also make nightmares more likely, so a medication review may be helpful.

*Type 8: Frequent night waking.* This concerns waking up in the night anxiously. Perhaps the person fears that someone is in the room or trying to get in. Perhaps they have been woken by a noise or a bad dream. Getting back to sleep after this kind of interruption can be tricky. Sleep becomes fragmented. The quality of the night’s rest is poorer. There are two methods to dial down the impact of these nighttime wakings. (Waking up for other reasons, such as needing to go to the bathroom, requires other solutions.) First, we want to reduce the chances of waking up in the first place. This means stacking the odds in favor of good-quality sleep: for example, by ensuring the day is busy so sleep pressure is high, cutting down on caffeinated drinks so the body is less alert; setting up the bedroom to minimize environmental reasons to wake up, building a firm foundation of calmness in the evening, and having a sleep window of the right length and timing. Second, a better response is needed when the person does wake up. In particular, we aim to de-catastrophe the waking and its associated fears. This can mean working on responses to paranoid fears or hallucinations. It can be helpful to have a card close by to remind the person that they are okay and it will pass or to plan a relaxation exercise. It might be that a limit is set on any time spent checking.

*Type 9: Psychotic experiences in the evening that delay sleep onset.* Some people find it hard to go to sleep because they hear or smell, or feel frightening or distracting things. They may also feel very vulnerable to harm from others. It can even feel as though sleep is risky. As a result, the person is on edge and wants to protect themselves, which is not conducive to sleep. There are two key messages that we convey to patients. First, that however much a person may want to be on guard, sleep is inevitable. It is not possible to stay awake all the time. Second, the person knows that sometimes they have relaxed their guard, slept, and have been safe. Therefore, it may be best to try to sleep as well as possible, to be in a better position to deal with the fears. We want to make sure that proper tiredness can come via a busy day, a wind-down routine, and going to bed and getting up at the right times. Hence, if a person is going to bed too early (Type 1), leading to a greater focus on the psychotic experiences, then we would likely address that issue first. We also want to try different ways of dealing with the fears and frightening or distracting, or angering experiences at night. We work with people to imagine different ways of reacting to fears and sensations at nighttime, assess the pros and cons of each way of reacting, choose the best options, and try them out to see which works best. The best techniques enable a person not to engage with the fears or sensations but instead to let them go and refocus attention elsewhere.

*Type 10: Days of very limited sleep (2–3 h per night or less) with periods of catch-up sleep.* A few patients sometimes have only very small amounts of sleep at night because the world feels so frightening. Sometimes they do not sleep at all. Nights are spent pacing, checking, worrying, and ruminating. The person can feel very vulnerable and like they could be harmed at any moment. There can be racing thoughts and intense energy. This is followed by a period of crashing and a long catch-up of sleep. It is an exhausting pattern and the quality of sleep is often poor. We emphasize to patients that, although one can be on guard for a few nights, it is impossible to keep that up indefinitely. The guard needs to come down sometimes. The best approach is to do that each night for a period of time so that quality sleep can occur. This will help the person cope with their fears the rest of the time. We seek to set an amount of sleep each night and work to make that feel as safe as possible. We develop a plan to deal with fears earlier in the evening and put in place a thorough evening wind-down routine.

### Going forward

Interest in sleep and psychosis has started to accelerate in recent years. Several obvious and achievable steps can be taken for patient benefit. However, surprisingly, few evaluations of the effects of sleep treatment for patients diagnosed with psychosis have taken place. This needs to change. We would stress that sleep interventions must be of a suitable length and intensity. We are currently engaged in Sleeping Better (ISRCTN71800376), two linked multi-center trials testing our sleep intervention for patients with diagnosed non-affective psychosis and patients at ultra-high risk of psychosis. We also recommend a research strategy of using session-by-session outcome data to identify typical treatment trajectories and their potential predictors, and to use that learning to improve treatment (Jenner et al., 2024). For all this work, it would be extremely worthwhile to develop – with people with lived experience – better measures of subjective sleep experiences. Current treatment approaches have excellent effects. However, further foundational work may bring even better treatments. A greater understanding of the causes of sleep difficulties for people with psychosis would be valuable, built on qualitative and quantitative methodologies, and linked to different profiles of sleep presentations. There may also be considerable merit in learning how to obtain quality sleep by linkage of the understanding of treatment techniques to sleep architecture. We see a future in which clinical services include routine assessment of sleep difficulties for patients with psychosis and provide sleep interventions as a key step in initial treatment plans.

## References

[r1] Bagautdinova, J., Mayeli, A., Wilson, J. D., Donati, F. L., Colacot, R. M., Meyer, N., … Ferrarelli, F. (2023). Sleep abnormalities in different clinical stages of psychosis: A systematic review and meta-analysis. JAMA Psychiatry, 80(3), 202–210. 36652243 10.1001/jamapsychiatry.2022.4599 PMC9857809

[r2] Baglioni, C., Altena, E., Bjorvatn, B., Blom, K., Bothelius, K., Devoto, A., … Riemann, D. (2020). The European Academy for Cognitive Behavioural Therapy for Insomnia: An initiative of the European Insomnia Network to promote implementation and dissemination of treatment. Journal of Sleep Research, 29(2), e12967. 31856367 10.1111/jsr.12967

[r3] Bagrowska, P., Pionke-Ubych, R., Majchrowicz, K., & Gawęda, Ł. (2022). The mediating effect of negative emotions on the relationship between subjective sleep quality and paranoia-like thoughts. Journal of Psychiatric Research, 145, 132–136. 34920163 10.1016/j.jpsychires.2021.12.003

[r4] Ballesio, A., Musetti, A., Zagaria, A., Manari, T., Filosa, M., & Franceschini, C. (2022). Depression and mania symptoms mediate the relationship between insomnia and psychotic-like experiences in the general population. Sleep Epidemiology, 2, 100019.

[r5] Blanchard, J. J., Andrea, A., Orth, R. D., Savage, C., & Bennett, M. E. (2020a). Sleep disturbance and sleep-related impairment in psychotic disorders are related to both positive and negative symptoms. Psychiatry Research, 286, 112857. 32087449 10.1016/j.psychres.2020.112857 PMC7416463

[r6] Blanchard, J. J., Savage, C. L., Orth, R. D., Jacome, A. M., & Bennett, M. E. (2020b). Sleep problems and social impairment in psychosis: A transdiagnostic study examining multiple social domains. Frontiers in Psychiatry, 11, 486. 32547433 10.3389/fpsyt.2020.00486 PMC7270336

[r7] Bootzin, R. R. (1972). Stimulus control treatment for insomnia. Proceedings of the American Psychological Association, 7, 395–396.

[r8] Borbély, A. A. (1982). A two process model of sleep regulation. Human Neurobiology, 1(3), 195–204. 7185792

[r9] Bradley, J., Freeman, D., Chadwick, E., Harvey, A. G., Mullins, B., Johns, L., … Waite, F. (2018). Treating sleep problems in young people at ultra-high risk of psychosis: A feasibility case series. Behavioural and Cognitive Psychotherapy, 46(3), 276–291. 29081329 10.1017/S1352465817000601 PMC5906720

[r10] Carney, C. E., Buysse, D. J., Ancoli-Israel, S., Edinger, J. D., Krystal, A. D., Lichstein, K. L., & Morin, C. M. (2012). The consensus sleep diary: Standardizing prospective sleep self-monitoring. Sleep, 35(2), 287–302. 22294820 10.5665/sleep.1642 PMC3250369

[r11] Carruthers, S. P., Brunetti, G., & Rossell, S. L. (2021). Sleep disturbances and cognitive impairment in schizophrenia spectrum disorders: a systematic review and narrative synthesis. Sleep Medicine, 84, 8–19. 34090012 10.1016/j.sleep.2021.05.011

[r12] Cederlöf, E., Holm, M., Taipale, H., Tiihonen, J., Tanskanen, A., Lähteenvuo, M., … Haaki, W. (2024). Antipsychotic medications and sleep problems in patients with schizophrenia. Schizophrenia Research, 267, 230–238. 38579432 10.1016/j.schres.2024.03.015

[r13] Chiu, V. W., Ree, M., Janca, A., Iyyalol, R., Dragovic, M., & Waters, F. (2018). Sleep profiles and CBT-I response in schizophrenia and related psychoses. Psychiatry Research, 268, 279–287. 30077955 10.1016/j.psychres.2018.07.027

[r14] Clarke, L., Chisholm, K., Cappuccio, F. P., Tang, N. K., Miller, M. A., Elahi, F., & Thompson, A. D. (2021). Sleep disturbances and the at risk mental state: A systematic review and meta-analysis. Schizophrenia Research, 227, 81–91. 32646803 10.1016/j.schres.2020.06.027

[r15] De Crescenzo, F., D’Alò, G. L., Ostinelli, E. G., Ciabattini, M., Di Franco, V., Watanabe, N., … Cipriani, A. (2022). Comparative effects of pharmacological interventions for the acute and long-term management of insomnia disorder in adults: a systematic review and network meta-analysis. The Lancet, 400(10347), 170–184. 10.1016/S0140-6736(22)00878-9 35843245

[r16] Edinger, J. D., Arnedt, J. T., Bertisch, S. M., Carney, C. E., Harrington, J. J., Lichstein, K. L., … Martin, J. L. (2021). Behavioral and psychological treatments for chronic insomnia disorder in adults: An American Academy of Sleep Medicine clinical practice guideline. Journal of Clinical Sleep Medicine, 17(2), 255–262. 33164742 10.5664/jcsm.8986 PMC7853203

[r17] Espie, C. (2021). Overcoming insomnia 2nd edition: A self-help guide using cognitive behavioural techniques. Robinson.

[r18] Formica, M. J. C., Fuller-Tyszkiewicz, M., Reininghaus, U., Kempton, M., Delespaul, P., De Haan, L., … & Hartmann, J. A. (2024). Associations between disturbed sleep and attenuated psychotic experiences in people at clinical high risk for psychosis. Psychological Medicine, 54, 2254–2263. 10.1017/S0033291724000400 PMC11413346 38450445

[r19] Freeman, D. (2024). Developing psychological treatments for psychosis. The British Journal of Psychiatry, 224(5), 147–149. 38652062 10.1192/bjp.2024.5 PMC11039552

[r20] Freeman, D., Brugha, T., Meltzer, H., Jenkins, R., Stahl, D., & Bebbington, P. (2010). Persecutory ideation and insomnia: Findings from the second British National Survey of Psychiatric Morbidity. Journal of Psychiatric Research, 44(15), 1021–1026. 20434169 10.1016/j.jpsychires.2010.03.018 PMC2977847

[r21] Freeman, D., Pugh, K., Vorontsova, N., & Southgate, L. (2009). Insomnia and paranoia. Schizophrenia Research, 108(1–3), 280–284. 19097752 10.1016/j.schres.2008.12.001 PMC2697325

[r22] Freeman, D., Sheaves, B., Goodwin, G. M., Yu, L. M., Nickless, A., Harrison, P. J., … & Espie, C. A. (2017). The effects of improving sleep on mental health (OASIS): A randomised controlled trial with mediation analysis. The Lancet Psychiatry, 4(10), 749–758. 28888927 10.1016/S2215-0366(17)30328-0 PMC5614772

[r23] Freeman, D., Stahl, D., McManus, S., Meltzer, H., Brugha, T., Wiles, N., & Bebbington, P. (2012). Insomnia, worry, anxiety and depression as predictors of the occurrence and persistence of paranoid thinking. Social Psychiatry and Psychiatric Epidemiology, 47, 1195–1203. 21928153 10.1007/s00127-011-0433-1

[r24] Freeman, D., Taylor, K. M., Molodynski, A., & Waite, F. (2019). Treatable clinical intervention targets for patients with schizophrenia. Schizophrenia Research, 211, 44–50. 31326234 10.1016/j.schres.2019.07.016

[r25] Freeman, D., Waite, F., Startup, H., Myers, E., Lister, R., McInerney, J., … Yu, L. M. (2015). Efficacy of cognitive behavioural therapy for sleep improvement in patients with persistent delusions and hallucinations (BEST): A prospective, assessor-blind, randomised controlled pilot trial. The Lancet Psychiatry, 2(11), 975–983. 26363701 10.1016/S2215-0366(15)00314-4 PMC4641164

[r26] Giles, J. J., Ling, I., McArdle, N., Bucks, R. S., Cadby, G., Singh, B., … Waters, F. (2022). Obstructive sleep apnea is treatable with continuous positive airway pressure in people with schizophrenia and other psychotic disorders. Schizophrenia Bulletin, 48(2), 437–446. 34581411 10.1093/schbul/sbab100 PMC8886585

[r27] Goldberg, L.R. (1990). An alternative “description of personality”: The big-five factor structure. Journal of Personality and Social Psychology, 59, 1216–1229. 2283588 10.1037//0022-3514.59.6.1216

[r28] Harvey, A. G. (2008). Sleep and circadian rhythms in bipolar disorder: seeking synchrony, harmony, and regulation. American Journal of Psychiatry, 165(7), 820–829. 18519522 10.1176/appi.ajp.2008.08010098

[r29] Harvey, A. G., & Buysse, D. J. (2017). Treating sleep problems: A transdiagnostic approach. Guilford Publications.

[r30] Harvey, A. G., Dong, L., Hein, K., Yu, S. H., Martinez, A. J., Gumport, N. B., … Buysse, D. J. (2021). A randomized controlled trial of the Transdiagnostic Intervention for Sleep and Circadian Dysfunction (TranS-C) to improve serious mental illness outcomes in a community setting. Journal of Consulting and Clinical Psychology, 89(6), 537. 34264701 10.1037/ccp0000650 PMC9377521

[r31] Harvey, A. G. & Sarfan, L. D. (2024). State of the science: The transdiagnostic intervention for sleep and circadian dysfunction. Behavior Therapy, 55, 1289–1302. 39443066 10.1016/j.beth.2024.02.007

[r32] Jenner, L., Payne, M., Waite, F., Beckwith, H., Diamond, R., Isham, L., … Freeman, D. (2024). Theory driven psychological therapy for persecutory delusions: trajectories of patient outcomes. Psychological Medicine, 54(15), 4173–4181. 39552390 10.1017/S0033291724002113 PMC11650162

[r33] Kalucy, M. J., Grunstein, R., Lambert, T., & Glozier, N. (2013). Obstructive sleep apnoea and schizophrenia–A research agenda. Sleep Medicine Reviews, 17(5), 357–365. 23528272 10.1016/j.smrv.2012.10.003

[r34] Kammerer, M. K., Mehl, S., Ludwig, L., & Lincoln, T. M. (2021). Sleep and circadian rhythm disruption predict persecutory symptom severity in day-to-day life: A combined actigraphy and experience sampling study. Journal of Abnormal Psychology, 130(1), 78. 33211503 10.1037/abn0000645

[r35] Kang, S. G., Lee, H. J., Jung, S. W., Cho, S. N., Han, C., Kim, Y. K., … Kim, L. (2007). Characteristics and clinical correlates of restless legs syndrome in schizophrenia. Progress in Neuro-Psychopharmacology and Biological Psychiatry, 31(5), 1078–1083. 17459547 10.1016/j.pnpbp.2007.03.011

[r36] Kaplan, K. A. (2020). Sleep and sleep treatments in bipolar disorder. Current Opinion in Psychology, 34, 117–122. 32203912 10.1016/j.copsyc.2020.02.001

[r37] Kasanova, Z., Hajdúk, M., Thewissen, V., & Myin-Germeys, I. (2020). Temporal associations between sleep quality and paranoia across the paranoia continuum: An experience sampling study. Journal of Abnormal Psychology, 129(1), 122. 31343182 10.1037/abn0000453

[r38] Kimhy, D., Ospina, L., Beck-Felts, K., Fakhoury, A., Mullins, A. E., & Varga, A. W. (2022). The impact of sleep on neurocognition and functioning in schizophrenia—is it time to wake-up?. Journal of Psychiatry and Brain Science, 7, e220001 35224206 10.20900/jpbs.20220001 PMC8880843

[r39] Koyanagi, A., & Stickley, A. (2015). The association between sleep problems and psychotic symptoms in the general population: A global perspective. Sleep, 38(12), 1875–1885. 26085291 10.5665/sleep.5232 PMC4667394

[r40] Krakow, B., Kellner, R., Pathak, D., & Lambert, L. (1995). Imagery rehearsal treatment for chronic nightmares. Behaviour Research and Therapy, 33(7), 837–843. 7677723 10.1016/0005-7967(95)00009-m

[r41] Lunsford-Avery, J. R., LeBourgeois, M. K., Gupta, T., & Mittal, V. A. (2015). Actigraphic-measured sleep disturbance predicts increased positive symptoms in adolescents at ultra high-risk for psychosis: a longitudinal study. Schizophrenia Research, 164(1–3), 15–20. 25818627 10.1016/j.schres.2015.03.013 PMC4409558

[r42] Ma, X. R., Song, G. R., Xu, X. B., Tian, T., & Chang, S. H. (2018). The prevalence of sleep disturbance and its socio‐demographic and clinical correlates in first‐episode individuals with schizophrenia in rural China. Perspectives in Psychiatric Care, 54(1), 31–38. 27861956 10.1111/ppc.12197

[r43] Miller, B. J., McEvoy, J. P., & McCall, W. V. (2023). Meta-analysis of clozapine and insomnia in schizophrenia. Schizophrenia Research, 252, 208–215. 36669344 10.1016/j.schres.2023.01.018

[r44] Morin, C. M., Khullar, A., Robillard, R., Desautels, A., Mak, M. S., Dang-Vu, T. T., … Carney, C. E. (2024). Delphi consensus recommendations for the management of chronic insomnia in Canada. Sleep Medicine, 124, 598–605. 39481275 10.1016/j.sleep.2024.09.038

[r45] Myers, E., Startup, H., & Freeman, D. (2011). Cognitive behavioural treatment of insomnia in individuals with persistent persecutory delusions: A pilot trial. Journal of Behavior Therapy and Experimental Psychiatry, 42(3), 330–336. 21367359 10.1016/j.jbtep.2011.02.004 PMC3566479

[r46] Ong, W. J., Tan, X. W., Shahwan, S., Satghare, P., Cetty, L., Ng, B. T., … Subramaniam, M. (2020). Association between sleep quality and domains of quality of life amongst patients with first episode psychosis. Health and Quality of Life Outcomes, 18, 1–9. 32349756 10.1186/s12955-020-01367-3 PMC7189457

[r47] Palagini, L., Manni, R., Liguori, C., De Gennaro, L., Gemignani, A., Fanfulla, F., … Ferini-Strambi, L. (2024). Evaluation and management of insomnia in the clinical practice in Italy: a 2023 update from the Insomnia Expert Consensus Group. Journal of Neurology, 271(4), 1668–1679. 38063870 10.1007/s00415-023-12112-3

[r48] Reed, Z. E., Jones, H. J., Hemani, G., Zammit, S., & Davis, O. S. (2019). Schizophrenia liability shares common molecular genetic risk factors with sleep duration and nightmares in childhood. Wellcome Open Research, 4. 10.12688/wellcomeopenres.15060.1 PMC6753602 31544153

[r49] Reeve, S., Nickless, A., Sheaves, B., & Freeman, D. (2018). Insomnia, negative affect, and psychotic experiences: Modelling pathways over time in a clinical observational study. Psychiatry Research, 269, 673–680. 30216919 10.1016/j.psychres.2018.08.090 PMC6215774

[r50] Reeve, S., Sheaves, B., & Freeman, D. (2019). Sleep disorders in early psychosis: Incidence, severity, and association with clinical symptoms. Schizophrenia Bulletin, 45(2), 287–295. 30202909 10.1093/schbul/sby129 PMC6403049

[r51] Reeve, S., Sheaves, B., & Freeman, D. (2021). Excessive sleepiness in patients with psychosis: An initial investigation. PLoS One, 16(1), e0245301. 33449971 10.1371/journal.pone.0245301 PMC7810297

[r52] Rehman, A., Gumley, A., & Biello, S. (2018). Sleep quality and paranoia: The role of alexithymia, negative emotions and perceptual anomalies. Psychiatry Research, 259, 216–222. 29080493 10.1016/j.psychres.2017.09.066

[r53] Riemann, D., Baglioni, C., Bassetti, C., Bjorvatn, B., Dolenc Groselj, L., Ellis, J.G., Espie, C.A., Garcia-Borreguero, D., Gjerstad, M., Gonçalves, M., Hertenstein, E., Jansson-Fröjmark, M., Jennum, P.J., Leger, D., Nissen, C., Parrino, L., Paunio, T., Pevernagie, D., Verbraecken, J., Weeß, H.G., Wichniak, A., Zavalko, I., Arnardottir, E.S., Deleanu, O.C., Strazisar, B., Zoetmulder, M., & Spiegelhalder, K. (2017). European guideline for the diagnosis and treatment of insomnia. Journal of Sleep Research, 26, 675–700. 28875581 10.1111/jsr.12594

[r54] Riemann, D., Benz, F., Dressle, R. J., Espie, C. A., Johann, A. F., Blanken, T. F., … Van Someren, E. J. (2022). Insomnia disorder: State of the science and challenges for the future. Journal of Sleep Research, 31(4), e13604. 35460140 10.1111/jsr.13604

[r55] Ritsner, M., Kurs, R., Ponizovsky, A., & Hadjez, J. (2004). Perceived quality of life in schizophrenia: relationships to sleep quality. Quality of Life Research, 13, 783–791. 15129888 10.1023/B:QURE.0000021687.18783.d6

[r56] Saper, C. B., Cano, G., & Scammell, T. E. (2005). Homeostatic, circadian, and emotional regulation of sleep. Journal of Comparative Neurology, 493(1), 92–98. 16254994 10.1002/cne.20770

[r57] Sarfan, L. D., Hilmoe, H. E., Gumport, N. B., & Harvey, A. G. (2023). The Transdiagnostic Intervention for Sleep and Circadian Dysfunction (TranS-C) in community mental health: Comorbidity and use of modules under the microscope. Cognitive and Behavioral Practice, 30(4), 692–706. 39429752 10.1016/j.cbpra.2022.03.007 PMC11488694

[r58] Scott, A. J., Rowse, G., & Webb, T. L. (2017). A structural equation model of the relationship between insomnia, negative affect, and paranoid thinking. PloS One, 12(10), e0186233. 29049381 10.1371/journal.pone.0186233 PMC5648154

[r59] Sheaves, B., Bebbington, P. E., Goodwin, G. M., Harrison, P. J., Espie, C. A., Foster, R. G., & Freeman, D. (2016). Insomnia and hallucinations in the general population: Findings from the 2000 and 2007 British Psychiatric Morbidity Surveys. Psychiatry Research, 241, 141–146. 27173659 10.1016/j.psychres.2016.03.055 PMC4922385

[r60] Sheaves, B., Freeman, D., Isham, L., McInerney, J., Nickless, A., Yu, L. M., … Barrera, A. (2018). Stabilising sleep for patients admitted at acute crisis to a psychiatric hospital (OWLS): An assessor-blind pilot randomised controlled trial. Psychological Medicine, 48(10), 1694–1704. 29108526 10.1017/S0033291717003191 PMC6088775

[r61] Sheaves, B., Holmes, E. A., Rek, S., Taylor, K. M., Nickless, A., Waite, F., … Freeman, D. (2019). Cognitive behavioural therapy for nightmares for patients with persecutory delusions (Nites): An assessor-blind, pilot randomized controlled trial. The Canadian Journal of Psychiatry, 64(10), 686–696. 31129983 10.1177/0706743719847422 PMC6783669

[r62] Sheaves, B., Isham, L., Bradley, J., Espie, C., Barrera, A., Waite, F., … Freeman, D. (2018). Adapted CBT to stabilize sleep on psychiatric wards: A transdiagnostic treatment approach. Behavioural and Cognitive Psychotherapy, 46(6), 661–675. 29615140 10.1017/S1352465817000789 PMC6141994

[r63] Sheaves, B., Onwumere, J., Keen, N., Stahl, D., & Kuipers, E. (2015). Nightmares in patients with psychosis: The relation with sleep, psychotic, affective, and cognitive symptoms. The Canadian Journal of Psychiatry, 60(8), 354–361. 26454557 10.1177/070674371506000804 PMC4542515

[r64] Stafford, A., Oduola, S., & Reeve, S. (2024). Sleep and socio-occupational functioning in adults with serious mental illness: A systematic review. Psychiatry Research, 116111. 39083962 10.1016/j.psychres.2024.116111

[r65] Taylor, M. J., Gregory, A. M., Freeman, D., & Ronald, A. (2015). Do sleep disturbances and psychotic-like experiences in adolescence share genetic and environmental influences? Journal of Abnormal Psychology, 124(3), 674. 25938536 10.1037/abn0000057 PMC4532318

[r66] Tonon, A. C., Nexha, A., Mendonça da Silva, M., Gomes, F. A., Hidalgo, M. P., & Frey, B. N. (2024). Sleep and circadian disruption in bipolar disorders: From psychopathology to digital phenotyping in clinical practice. Psychiatry and Clinical Neurosciences, 78(11), 654–666. 39210713 10.1111/pcn.13729 PMC11804932

[r67] Waite, F., Bradley, J., Chadwick, E., Reeve, S., Bird, J. C., & Freeman, D. (2018). The experience of sleep problems and their treatment in young people at ultra-high risk of psychosis: A thematic analysis. Frontiers in Psychiatry, 9, 375. 30197607 10.3389/fpsyt.2018.00375 PMC6117428

[r68] Waite, F., Černis, E., Kabir, T., Iredale, E., Johns, L., Maughan, D., … Freeman, D. (2023). A targeted psychological treatment for sleep problems in young people at ultra-high risk of psychosis in England (SleepWell): a parallel group, single-blind, randomised controlled feasibility trial. The Lancet Psychiatry, 10(9), 706–718. 37562423 10.1016/S2215-0366(23)00203-1

[r69] Waite, F., Evans, N., Myers, E., Startup, H., Lister, R., Harvey, A. G., & Freeman, D. (2016). The patient experience of sleep problems and their treatment in the context of current delusions and hallucinations. Psychology and Psychotherapy: Theory, Research and Practice, 89(2), 181–193. 10.1111/papt.12073 PMC4879509 26285922

[r70] Waite, F., Evans, S., Rebello, A., Sharpe, T., Otaiku, J., Iredale, E., Kabir, T., Černis, E., & Freeman, D. (manuscript submitted for publication). Sleep disruption and its psychological treatment in young people at-risk of psychosis: A peer methods qualitative evaluation. 10.1111/bjc.70002 PMC13456408 40619649

[r71] Waite, F., Myers, E., Harvey, A. G., Espie, C. A., Startup, H., Sheaves, B., & Freeman, D. (2016). Treating sleep problems in patients with schizophrenia. Behavioural and Cognitive Psychotherapy, 44(3), 273–287. 26751571 10.1017/S1352465815000430 PMC4855992

[r72] Waite, F., Sheaves, B., Isham, L., Reeve, S., & Freeman, D. (2020). Sleep and schizophrenia: From epiphenomenon to treatable causal target. Schizophrenia Research, 221, 44–56. 31831262 10.1016/j.schres.2019.11.014 PMC7327507

[r73] Wang, D., Ma, Z., Scherffius, A., Liu, W., Bu, L., Sun, M., & Fan, F. (2023). Sleep disturbance is predictive of psychotic-like experiences among adolescents: A two-wave longitudinal survey. Sleep Medicine, 101, 296–304. 36470165 10.1016/j.sleep.2022.11.011

[r74] Waters, F., Chiu, V., Atkinson, A., & Blom, J. D. (2018). Severe sleep deprivation causes hallucinations and a gradual progression toward psychosis with increasing time awake. Frontiers in Psychiatry, 9, 350067. 10.3389/fpsyt.2018.00303 PMC6048360 30042701

[r75] Weber, F. C., Danker-Hopfe, H., Dogan-Sander, E., Frase, L., Hansel, A., Mauche, N., … Wetter, T. C. (2022). Restless legs syndrome prevalence and clinical correlates among psychiatric inpatients: A multicenter study. Frontiers in Psychiatry, 13, 846165. 35370821 10.3389/fpsyt.2022.846165 PMC8967168

[r76] Winkelman, J. W. (2001). Schizophrenia, obesity, and obstructive sleep apnea. Journal of Clinical Psychiatry, 62(1), 8–11. 10.4088/jcp.v62n0103 11235938

